# Impact of autologous serum on an in vitro granuloma model to study the dynamics of *Mycobacterium tuberculosis* infection

**DOI:** 10.5588/ijtldopen.24.0691

**Published:** 2025-05-12

**Authors:** C. Bourg, E. Hodille, F. Ader, O. Dumitrescu, C. Genestet

**Affiliations:** ^1^ CIRI - Centre International de Recherche en Infectiologie, Ecole Normale Supérieure de Lyon, Université Claude Bernard Lyon-1, Inserm U1111, CNRS UMR5308, Lyon, France;; ^2^ Hospices Civils de Lyon, Institut des Agents Infectieux, Laboratoire de bactériologie, Lyon, France;; ^3^ Hospices Civils de Lyon, Service des Maladies infectieuses et tropicales, Lyon, France;; ^4^ Université Lyon 1, Facultés de Médecine et de Pharmacie de Lyon, Lyon, France.

**Keywords:** tuberculosis, granuloma-like structures, complement system, host-pathogen interaction, immune response

Dear Editor,

TB remains a significant global health challenge, with *Mycobacterium tuberculosis* (Mtb) infecting nearly a quarter of the world's population.^1^ A common pathological feature of TB is the granuloma, a dynamic immune structure comprised of various immune cells (including macrophages and lymphocytes) that arises in response to infection. It acts as a barrier to prevent the spread of bacteria while its immune cells produce cytokines to eliminate the infection. Despite this protective role, granulomas often fail to completely eradicate the bacteria, creating a niche where Mtb can persist in a latent state. This dual nature, simultaneously containing the bacteria and enabling its survival, reflects the delicate balance between host defences and bacterial persistence mechanisms. Understanding granuloma formation and function is therefore essential for elucidating TB progression, as it encapsulates the complex interplay between immune responses and pathogen persistence.^2^

In vitro granuloma models have been instrumental in advancing our understanding of TB mechanisms and could be envisioned as a preclinical model to evaluate new anti-TB strategies.^3^ However, many of these models rely on inactivated commercial human AB serum (CHS) to supplement the cell culture medium,^4,5^ which is pooled from multiple donors to minimize variability, but which may not fully replicate the complexity of the native immune environment. Autologous serum – either native autologous serum (NAS) or heat-inactivated autologous serum (HIAS) – has been suggested to better support certain immune cell functions, such as cytokine secretion, lymphocyte proliferation and complement activity (Figure panel A).^6,7^ These factors could potentially influence granuloma dynamics and immune polarization.^8,9^ However, the role of autologous serum in *in vitro* granuloma formation, and its impact on immune response dynamics, remains to be investigated. To address this, we assessed the dynamics of Mtb infection and granuloma formation using a previously developed protocol.^3^

**Figure.**
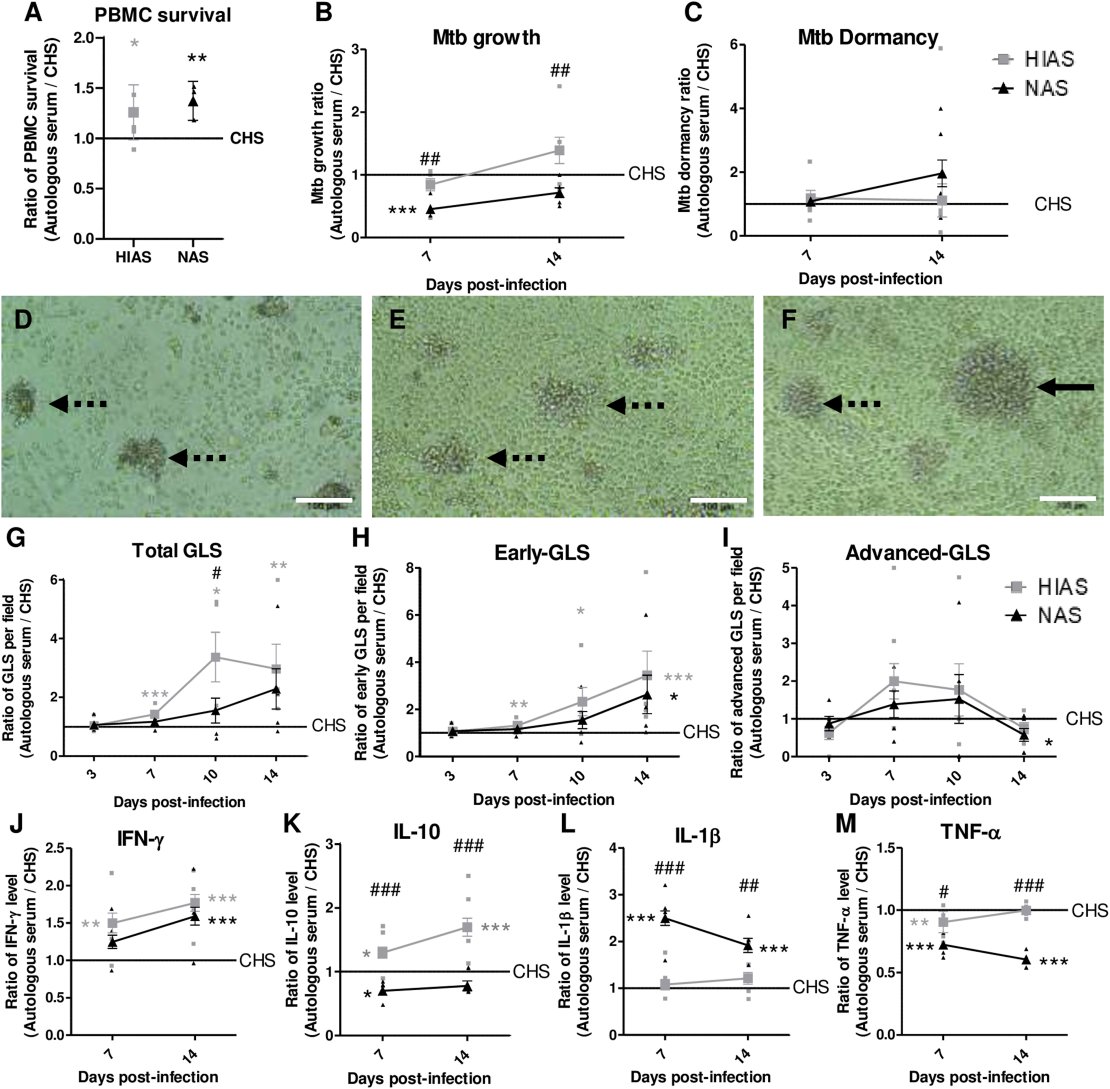
Impact of autologous serum on an in vitro granuloma model to study Mtb infection dynamics. **A:** PBMC survival after 7 days of culture in culture media supplemented with commercial human AB serum (CHS), heat-inactivated autologous serum (HIAS) or native autologous serum (NAS). **B–M**: PBMC were infected with the H37Ra Mtb reference strain at a multiplicity of infection of 1:10 (bacteria:cell), in presence of CHS (used as a control to normalise the results), HIAS (grey square) or NAS (black triangle), to induce spontaneous formation of in vitro granulomas. At 7- and 14-days post-infection, after granuloma disruption and cellular lysis, (**B**) Mtb growth was evaluated by CFU counting and (**C**) Mtb dormancy was assessed by quantifying differentially culturable bacteria in the presence or absence of stationary-phase filtered culture supernatant of Mtb containing resuscitation-promoting factors using the mycobacterial growth incubator tube (MGIT) time to positivity system, reflecting bacterial growth.^3^ **D–F**: Representative bright field images of in vitro granulomas at 7-days post-infection from a representative donor. Dotte arrows show early grade GLS and complete one show advanced grade GLS. Scale bar = 100μm. **G–I**: At 3-, 7-, 10- and 14-days post-infection, the dynamics of the total (**D**), early grade (**E**) and advanced grade (**F**) granuloma-like structure (GLS) observed per field was assessed by optical microscopy. Early grades correspond to start of granuloma formation, involving mainly monocytes/macrophages, with diameter <100µm. Advanced grades correspond to multilayer structures involving monocytes/macrophages surrounded by lymphocytes, with diameter >100µm ^10^. **J**: IFN-γ; **K**: IL-10; **L**: IL-1β and **M**: TNF-α release in cell culture supernatant was evaluated at 7- and 14-days post-infection, using Simple Plex Cytokine Screening Panel (Bio-techne, Minneapolis, USA) on a EllaTM (Bio-techne). Small symbols represent the mean for each patient, while large symbols indicate the overall mean. Values for each condition are the mean ± standard error of measurement of four healthy donors and two independent experiments. Means were compared using One-Way ANOVA followed by Bonferroni correction. Grey * represent the comparison between HIAS and CHS; black * represent the comparison between NAS and CHS; # represent the comparison between HIAS and NAS. *p<0.05, **p<0.01, ***p<0.001, ****p<0.0001.

Peripheral blood mononuclear cells (PBMC) from four IGRA TB-negative, medication-free healthy donors were infected with the avirulent Mtb strain H37Ra to induce spontaneous formation of in vitro granulomas, as previously shown.^3^ The experimental setup, performed in biological duplicate, compared the effect of culture media supplementation with CHS, HIAS, and NAS on bacterial growth and dormancy, the formation of granuloma-like structures (GLS), and immune response dynamics over 14-days post-infection (dpi). Bacterial growth was evaluated by CFU counting, and bacterial dormancy was assessed by quantifying differentially culturable bacteria in presence or absence of stationary-phase filtered culture supernatant of Mtb containing resuscitation-promoting factors. GLS were quantified by optical microscopy, and immune response dynamics was analysed by measuring cytokine levels in culture supernatants^3,10^ All results were normalized on those obtained for each donor upon infection in presence of CHS, as it is the widely used condition.

Our results show that infection in the presence of different serum types had a modest impact on bacterial growth dynamics (Figure panel B). Growth was comparable upon infection in the presence of CHS and HIAS, whereas infection in the presence of NAS significantly reduced bacterial replication, with ratios of 0.45±0.042 and 0.71±0.077 compared to CHS at 7- and 14-dpi, respectively. Despite this, no significant difference was observed regarding induction of Mtb dormancy (Figure panel C). Regarding GLS formation (Figure panels D–I), infection in the presence of HIAS and NAS resulted in enhanced GLS formation compared to CHS, with up to 2.96±0.83 and 2.29±0.68 times more GLS per field observed at 14-dpi, respectively (Figure panel G). This increase was primarily driven by a significantly higher formation of early-GLS (up to 3.44±1.02 time more early-GLS per field at 14-dpi; Figure panel H). The marked increase in GLS numbers observed over time with autologous serum, whether HIAS or NAS, is likely driven by improved PBMC survival in its presence. Our data (Figure panel A) show that autologous serum enhances PBMC viability, providing a favourable environment for GLS formation. This is supported by prior findings showing improved PBMC survival with autologous plasma supplementation.^11^

This higher formation of GLS upon infection in presence of autologous serum, whether HIAS or NAS, was associated with a higher production of IFN-γ compared to CHS (1.77±0.51 and 1.59±0.53 at 14-dpi, respectively; Figure panel J). However, the cytokine profiles diverged between these conditions. For infection in presence of HIAS, the parallel increase in IL-10 production suggested the preservation of a balanced pro- and anti-inflammatory response. In contrast, infection in the presence of NAS was characterized by reduced IL-10 production (down to a ratio of 0.70±0.27 at 7-dpi), indicative of an intensified pro-inflammatory response (Figure panel K). This heightened response was further evidenced by significantly elevated IL-1β levels (2.50±0.69 at 7 dpi and 1.92±0.68 at 14 dpi), despite a slight reduction in TNF-α production (Figure panel L–M). The active complement system in NAS likely contributes to this enhanced pro-inflammatory profile. The complement system is critical in modulating immune responses during Mtb infection, promoting inflammasome activation and IL-1β secretion.^12,13^ Additionally, the complement system enhances Mtb phagocytosis and intracellular killing by macrophages, and its dysfunction has been linked to increased susceptibility to TB in patients with type 2 diabetes mellitus.^13,14^ This mechanism could explain the reduced bacterial load observed upon infection in presence of NAS (Figure panel B). Overall, these distinct immune profiles underscore the differing impacts of the serum used on immune response dynamics.

Our findings suggest that the type of serum used during infection has significant impact on both bacterial growth dynamics and GLS formation and the associated immune response. Notably, the use of native serum, containing the complement system with a key-role in immune modulation and bacterial clearance, enables better control of the bacterial load, whilst the autologous serum favours GLS formation. This model offers potential as a preclinical tool for evaluating TB therapies, where physiological relevance is important, particularly with cells from TB patients. Incorporating the patient’s own serum allows the inclusion of host-specific components, such as cytokines, immune factors and the complement system, which are essential to accurately reflecting the patient’s immune environment to mimic in vivo conditions, thereby advancing our ability to test and develop targeted therapies. Despite these insights, further exploration is required to elucidate the precise mechanisms underlying these immune dynamics, including the roles of the complement system. Additionally, it is well-established that the H37Ra strain, used in this study, induces a type I immune response, unlike virulent Mtb strains which favor a type II response.^15^ Characterizing this model with virulent clinical Mtb strains will be critical for advancing its relevance as a preclinical tool.

In conclusion, autologous serum supports sustained GLS formation and offers a more representative environment for studying host-pathogen interactions compared to commercial human serum. Although additional characterization is needed, our results underscore the importance of autologous serum for developing physiologically relevant in vitro granuloma models.
